# MxA mRNA decrease preceding NAb detection in IFNβ‐treated MS patients

**DOI:** 10.1002/brb3.644

**Published:** 2017-02-09

**Authors:** Jana Libertinova, Eva Meluzinova, Vaclav Matoska, Miroslav Zajac, Ivana Kovarova, Eva Havrdova, Dana Horakova, Ales Tomek, Petr Marusic, Martin Bojar

**Affiliations:** ^1^ Department of NeurologyCharles University2nd Faculty of Medicine and Motol University HospitalPragueCzech Republic; ^2^ Laboratory of Molecular DiagnosticsNa Homolce HospitalPragueCzech Republic; ^3^ Department of Medical Microbiology, Charles University2nd Faculty of Medicine and Motol University HospitalPragueCzech Republic; ^4^ Department of Neurology and Center of Clinical NeuroscienceCharles UniversityFirst Faculty of Medicine and General University HospitalPragueCzech Republic

**Keywords:** bioactivity, interferon beta, multiple sclerosis, MxA, MxA mRNA, neutralizing antibodies

## Abstract

**Background:**

Multiple sclerosis (MS) patients treated with interferon beta (IFNβ) are at risk of a declining response to treatment because of the production of IFNβ‐neutralizing antibodies (NAbs). The expression of Myxovirus resistance protein A (MxA) mRNA is regarded as a marker of IFNβ bioactivity.

**Aims:**

The aim of this study was to analyze the kinetics of MxA mRNA expression during long‐term IFNβ treatment and assess its relationship to NAb production.

**Methods:**

A prospective, observational, open‐label, non‐randomized study was designed in multiple sclerosis patients starting IFNβ treatment. NAbs and MxA mRNA were monitored every six months.

**Results:**

119 patients were consecutively enrolled and 107 were included in the final analysis. Both the presence of NAbs and a decrease in MxA mRNA below the cut off were revealed in 15 patients, however, in six patients (40%) positivity for NAbs was preceded by the decrease in MxA mRNA. In addition, a further six patients showing a decline in MxA mRNA did not have detectable NAbs.

**Conclusion:**

Our data indicate that quantification of MxA mRNA is a more sensitive identifier of loss of IFNβ efficacy than the NAb positivity.

## 1 Introduction

**Figure 1:**
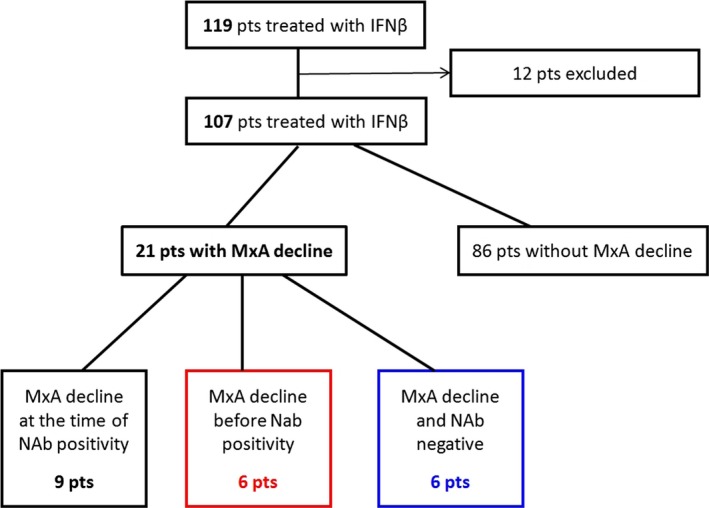
Kinetics of IFNβ bioactivity markers (NAbs, MxA mRNA) during the follow‐up period (24 months)

Interferon β (IFNβ) is one of the first‐line treatments for patients with clinically isolated syndrome or relapsing remitting multiple sclerosis (MS). The responsiveness to this treatment may be lost because of the production of neutralizing antibodies (NAbs), which prevent the interaction between IFNβ and its receptor. Myxovirus resistance protein A (MxA) is a downstream gene product of IFNβ and is used as a marker of the biological activity of IFNβ. The level of MxA mRNA quantifies the IFNβ biological response (Bertolotto et al., 2001) and there is clear evidence that lack of MxA mRNA indicates a completely attenuated response to IFNβ (Hesse, Sellebjerg, & Sørensen, 2009). The aim of our study was to assess the long‐term kinetics of these IFNβ bioactivity markers (NAbs, MxA mRNA), specifically focusing on the temporal appearance of NAbs and the decline in MxA mRNA (Figure 1).

## 2 Methods

A multicentre, prospective, observational, open‐label, non‐randomized study in MS patients starting IFNß treatment was performed. Patient enrolment started in June 2011 and was completed in January 2013, in total, 119 patients were enrolled. The duration of the follow‐up was 24 months. Patients were monitored every six months for the assessment of MxA mRNA expression and for the determination of NAbs. Blood samples were obtained 12 ± 3 hr after the last IFNß injection, in the absence of signs of infection. MxA mRNA was assessed by real‐time PCR in peripheral blood mononuclear cells. Blood samples were collected in EDTA tubes or TEMPUS Blood RNA tubes (ThermoFisher scientific), RNA extraction from EDTA collection tubes was performed with the QIAmp RNA blood Mini Kit (Quiagen), isolation from TEMPUS tubes was done using Tempus Spin RNA isolation kits (Applied Biosytems/ ThermoFisher scientific). The RNA concentrations in isolates were measured by a DeNovix D11‐ Spectrophotometer. Subsequent cDNA transcription and qPCR was done using EXPRESS One‐Step SYBR^®^ GreenER^™^ Universal Kits (Invitrogen/ Thermo Fisher Scientific) according to the manufacturer's protocol. PCR primers have been published elsewhere (Bertolotto et al., 2001). Reactions were performed on Rotor‐Gene (Corbett Research) and Rotor‐Gene Q (Quiagen) in duplicate. We set up a comparative quantification assay (Livak & Schmittgen, 2001) to assess the expression of MxA mRNA relative to GAPDH (glyceraldehyde‐3‐phosphate dehydrogenase) expression (according to the Rotor‐Gene and Rotor‐Gene Q manual “Comparative Quantification”). The cut off for MxA mRNA was established for our laboratory and the general Czech population. In cases of repeated MxA mRNA decrease below the cut off, we performed an MxA induction test with comparison of MxA levels pre‐ and post‐injection (van der Voort et al., 2009).

NAbs were determined using the antiviral cytopathic effect assay (Kawade, Finter, & Grossberg, 2003). Patients with positivity for NAbs and/or a decrease in MxA mRNA below the cut off were identified in the cohort and the time of appearance of both markers was compared.

The study protocol was approved by the Ethics Committee of Motol University Hospital and each participating patient gave informed consent.

### 2.1 Statistical analysis

All statistical analyses were performed using IBM SPSS Statistics 22 software – IBM Corporation, Armonk, New York, NY, USA (RRID:SCR_002865) and GraphPad Prism software 4.0 – GraphPad Software, San Diego, CA, USA (RRID:SCR_002798).

The cut off value for MxA mRNA was set to reflect an optimal reaction to IFNβ using the mean MxA value of control samples plus two standard deviations. An MxA mRNA value lower than this cut off in IFNβ‐treated patients was interpreted as inefficacy of the IFNβ treatment.

## 3 Results

In 40% of patients (six out of 15) demonstrating both NAb positivity and an MxA mRNA decrease below the cut off, the decrease in MxA mRNA was detected either six or 12 months earlier than the NAb positivity. This was mainly observed in patients treated with IFNβ‐1b s.c. (five patients), but also, in one patient treated with IFNβ‐1a s.c. (Table 1). Six patients presented with MxA mRNA below the cut off, but no NAb positivity over the whole follow‐up period. In two patients, NAbs and the decrease in MxA mRNA were not detected until month 24 (Figure 1).

**Table 1:** Time of occurrence of MxA mRNA decrease below the cut off value and the first detection of NAb positivity during the 24‐month follow‐up period

Patient (sex, age)	IFNβ	MxA↓	NAb+
M, 40	1b s.c.	M6	M6
M, 28	1b s.c.	**M6**	**M12**
M, 42	1b s.c.	**M6**	**M18**
M, 27	1b s.c.	**M6**	**M12**
F, 40	1b s.c.	M12	M12
F, 22	1b s.c.	M12	M12
F, 34	1b s.c.	**M12**	**M18**
F, 40	1b s.c.	**M12**	**M18**
F, 57	1a s.c.	M12	M12
M, 30	1a s.c.	M12	M12
F, 27	1a s.c.	M12	M12
F, 49	1a s.c.	**M12**	**M18**
F, 59	1a s.c.	M18	M18
F, 24	1a s.c.	M24	M24
M,19	1a s.c.	M24	M24

MxA↓ – MxA mRNA falling below the cut off value; NAb+ – NAb positivity. Timepoints of Nab+ /MxA↓ are given in months since initiation of IFNβ treatment (M6, M12, M18, M24). Cases where MxA↓ preceded NAbs+ are in bold.

## 4 Discussion

Our results indicate that the decrease in MxA mRNA is a more reliable marker of IFNβ treatment efficacy than the NAb positivity alone. The decline in MxA mRNA can precede the detection of NAbs by several months. Our observations support the hypothesis that once the patient has started IFNβ treatment, recurrently low MxA mRNA expression is predictive of NAb development. It is feasible that the early decrease in MxA mRNA could be caused by IFNβ binding antibodies (BAbs) that are detectable in the first months of IFNβ therapy. In some cases, BAbs have been shown to cause a loss of bioactivity, but not neutralizing activity in the CPE assay, thus these antibodies are not classed as NAbs (Gilli et al., 2007). However, it has been proposed that BAb titres may be a predictive tool for future NAb development (Hegen et al., 2014).

Another explanation for the decrease in MxA mRNA preceding NAb detection could be insufficient sensitivity of the CPE assay. Providing there is optimal methodological standardization, the CPE has a defined sensitivity limit of 5 TRU/ml and a titre of 20 TRU/ml is considered positive for NAbs, although mostly with a low clinical significance (Kawade et al., 2003). However, the effect of NAbs on the bioactivity of IFNβ is variable (Bertolotto, 2015) and there is a theoretical possibility that the biological activity could also be  reduced in patients with very low NAb titres (lower than 5 TRU/ml).

In several patients with a drop in MxA mRNA, NAbs were not detected at all during the follow‐up period. It is possible that our follow‐up period was too short to detect NAbs in all cases, which is supported by two cases not presenting with NAb positivity until month 24. Other explanations for non‐antibody mediated abolished biological activity have been reported, for example, patient non‐compliance and saturation of the IFN receptor (Gilli et al., 2007).

Nevertheless, in our opinion, it is the level of MxA mRNA expression and not NAb positivity that should be used as a primary tool for monitoring IFNβ treatment efficacy in MS patients. A lack of MxA mRNA expression strongly correlates with a loss of IFNβ therapy efficacy, regardless of NAb positivity. The current clinical recommendation is that IFNβ treatment is continued until NAbs are detected (Polman et al., 2010; Sørensen et al., 2005), however, our study reveals that MxA mRNA decline can precede NAb detection by six‐12 months. Thus, patients may be receiving an inefficacious treatment for a long period during which time their condition is, in effect, not being treated, which can lead to a high risk of relapse. Thus, our data support the implementation of routine quantification of MxA mRNA levels, with the caveat that repeated MxA mRNA measurements should be performed as the MxA level can fluctuate and the MxA mRNA induction test should be routinely used to confirm the drop in MxA mRNA.

## Conflicts of Interest

Tomek, Marusic and Bojar declare no conflict of interest. Libertinova received compensation for travel and speaker honoraria from Merck Serono, Biogen Idec, Novartis, Bayer Schering and Teva. Meluzinova received speaker honoraria, compensation for travel and consultant fees from Biogen Idec, Novartis, Merck Serono, Genzyme, Bayer Schering and Teva. Matoska and Zajac declare that there is no conflict of interest. Kovarova received compensation for travel and speaker honoraria from Novartis, Biogen Idec, Merck Serono, Bayer Schering and Teva. Havrdova received speaker honoraria and consultant fees from Biogen Idec, Merck Serono, Novartis, Genzyme, Teva, Actelion and Receptos, as well as support for research activities from Biogen Idec and Merck Serono. Horakova received compensation for travel, speaker honoraria and consultant fees from Biogen Idec, Novartis, Merck Serono, Bayer Schering and Teva, as well as support for research activities from Biogen Idec.
